# Rapid and efficient genetic manipulation of gyrencephalic carnivores using *in utero* electroporation

**DOI:** 10.1186/1756-6606-5-24

**Published:** 2012-06-20

**Authors:** Hiroshi Kawasaki, Lena Iwai, Kaori Tanno

**Affiliations:** 1 Department of Molecular and Systems Neurobiology, Graduate School of Medicine, The University of Tokyo, Hongo 7-3-1, Bunkyo-ku, Tokyo, 113-0033, Japan; 2 Global COE Program "Comprehensive Center of Education and Research for Chemical Biology of the Diseases", The University of Tokyo, Bunkyo-ku, Tokyo, 113-0033, Japan; 3 PRESTO, Japan Science and Technology Agency, Chiyoda-ku, Tokyo, 102-0076, Japan

**Keywords:** Ferrets, Cerebral cortex, *in utero* electroporation

## Abstract

**Background:**

Higher mammals such as primates and carnivores have highly developed unique brain structures such as the ocular dominance columns in the visual cortex, and the gyrus and outer subventricular zone of the cerebral cortex. However, our molecular understanding of the formation, function and diseases of these structures is still limited, mainly because genetic manipulations that can be applied to higher mammals are still poorly available.

**Results:**

Here we developed and validated a rapid and efficient technique that enables genetic manipulations in the brain of gyrencephalic carnivores using *in utero* electroporation. Transgene-expressing ferret babies were obtained within a few weeks after electroporation. GFP expression was detectable in the embryo and was observed at least 2 months after birth. Our technique was useful for expressing transgenes in both superficial and deep cortical neurons, and for examining the dendritic morphologies and axonal trajectories of GFP-expressing neurons in ferrets. Furthermore, multiple genes were efficiently co-expressed in the same neurons.

**Conclusion:**

Our method promises to be a powerful tool for investigating the fundamental mechanisms underlying the development, function and pathophysiology of brain structures which are unique to higher mammals.

## Background

Higher mammals such as carnivores and primates have highly developed brain structures, such as the ocular dominance columns (ODC) in the visual cortex and the gyrus and outer subventricular zone (OSVZ) of the cerebral cortex, which are unique to higher mammals. Uncovering the physiological importance and developmental processes of these structures using higher mammals would lead to the ultimate goal of understanding the human brain and its diseases. Although there have been extensive anatomical and electrophysiological investigations, our molecular understanding of the formation, function and pathophysiology of these structures is still limited. This is mainly because mice do not have these structures, and rapid and efficient genetic manipulations that can be applied to higher mammals are still poorly available.

Recently, several groups including us have identified molecules with intriguing expression patterns in the cerebral cortex and the thalamus of higher mammals such as ferrets and monkeys [1-6]. To investigate the functions of these molecules in the brain of higher mammals, we decided to establish a gene manipulation technique for higher mammals. Although *in utero* electroporation is well-known to be a useful technique to express genes of interests in the living rodent brain [7-11], successful application of *in utero* electroporation in higher mammals has not been reported. Here, we developed a rapid and efficient procedure of *in utero* electroporation for gyrencephalic carnivore ferrets. Using our procedure, genes of interest can be rapidly and efficiently expressed in the living ferret brain. Our method promises to be a powerful tool for investigating the fundamental mechanisms underlying the development, function and pathophysiology of brain structures which are unique to higher mammals.

## Results

**Figure 1:**
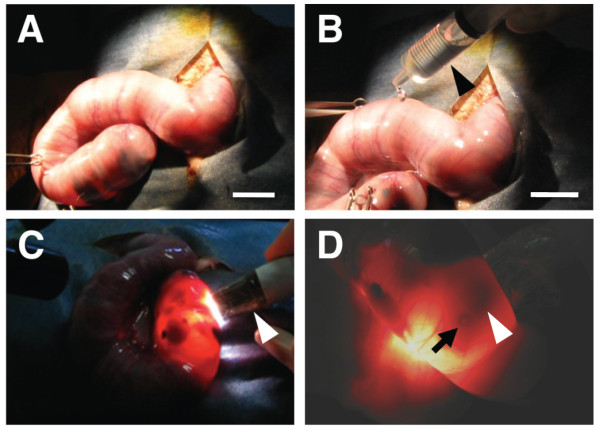
**Procedure for* in utero *electroporation in ferrets.** (**A**) A pregnant ferret was anesthetized at E36, and the right uterus was exposed. (**B**) The exposed uterus was kept wet with PBS by using a syringe (arrowhead). Note that the embryo within the uterus was invisible under epi-illumination. (**C**) Visualizing ferret embryos in the uterus using transmitted light derived from an optical fiber cable (arrowhead). Performing *in utero* electroporation in a dark room is helpful for visualizing ferret embryos in the uterus. (**D**) A ferret embryo in the uterus revealed with transmitted light. The pigmented iris and injected plasmid solution containing Fast Green were visible (arrow and arrowhead, respectively). Scale bars, 2 cm.

We first tried to apply the *in utero* electroporation procedure for mice to ferrets. We anesthetized pregnant ferrets between embryonic days 36 and 40 (E36-E40) and opened their abdominal cavities. When the uterine horns were exposed, we noticed that the uterus of pregnant ferrets was much bigger than that of pregnant mice; the diameter of the ferret uterus was about 2-3 cm at this age (Figure 1, A and B). It was often difficult to see where embryos were and to identify the location of ferret embryos in the uterus even when using epi-illumination, presumably because the uterine wall of ferrets was thicker than that of mice (Figure 1, A and B). To overcome this problem, we first decided to try *exo utero* electroporation, which is used for mouse embryos and involves cutting uterine muscles while leaving the amniotic membrane intact [12]. Cutting the uterine muscles enabled us to easily identify ferret embryos in the amniotic cavity. However, after a couple of days, when we examined the condition of the embryos, we found that they were unhealthy or had died, presumably because the large size of the placenta of ferrets had resulted in its being damaged when the uterine muscles were cut.

To visualize ferret embryos in the uterus, we next modified our illumination settings. We illuminated the ferret uterus using transmitted light and found that ferret embryos became clearly visible through the uterine wall (Figure 1, C and D). The pigmented iris was visible (Figure 1D, arrow), and the location of the iris enabled us to assume the location of the lateral ventricle and to inject plasmid solutions into the lateral ventricle using a pulled glass micropipette (Figure 1D, arrowhead).

**Figure 2:**
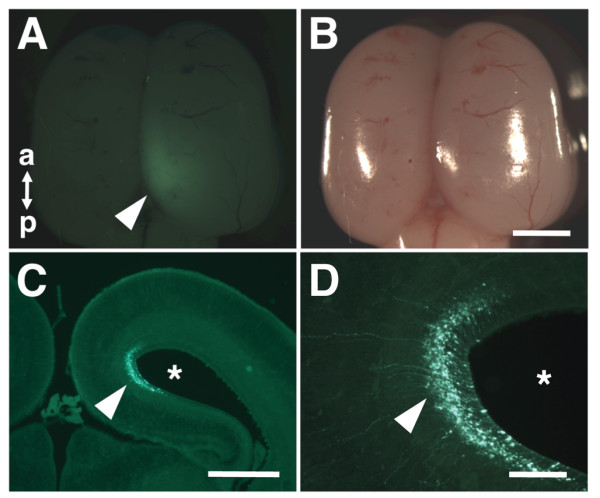
**GFP signals in the ferret cerebral cortex induced by* in utero *electroporation.** pCAG-GFP was introduced to the ferret cerebral cortex by using *in utero* electroporation (50 V, 50 ms) at E40, and ferret embryos were dissected 2 days later. Macroscopic dorsal views of the ferret brain (**B**) and its GFP fluorescence (**A**) are shown. Note that GFP signals were visible on the brain surface (arrowhead). a, anterior; p, posterior. Lower (**C**) and higher (**D**) magnification images of the coronal section of the electroporated brain. GFP-positive cells extending their processes toward the pial surface were observed in the cerebral cortex (arrowheads). Asterisks, the lateral ventricle. Scale bars, 2 mm (**B**), 1 mm (**C**), 200 μm (**D**).

We next turned to determining the proper voltage for electric pulses. We first tried 40-50 V (50 ms duration, 1 s interval, 5 times), which is commonly used for *in utero* electroporation for mice, and dissected ferret embryos a few days later. We successfully observed GFP fluorescence on the surface of the cerebral cortex, although it was not very strong (Figure 2, A and B). We prepared the section of the ferret brain and found GFP-positive areas around the lateral ventricle (Figure 2C, arrowhead). Higher magnification images showed several GFP-positive cells extending their processes toward the pial surface in the cerebral cortex (Figure 2D, arrowhead). These results suggest that *in utero* electroporation is practically feasible for ferrets, but the transfection efficiency is not good enough under these conditions.

**Figure 3:**
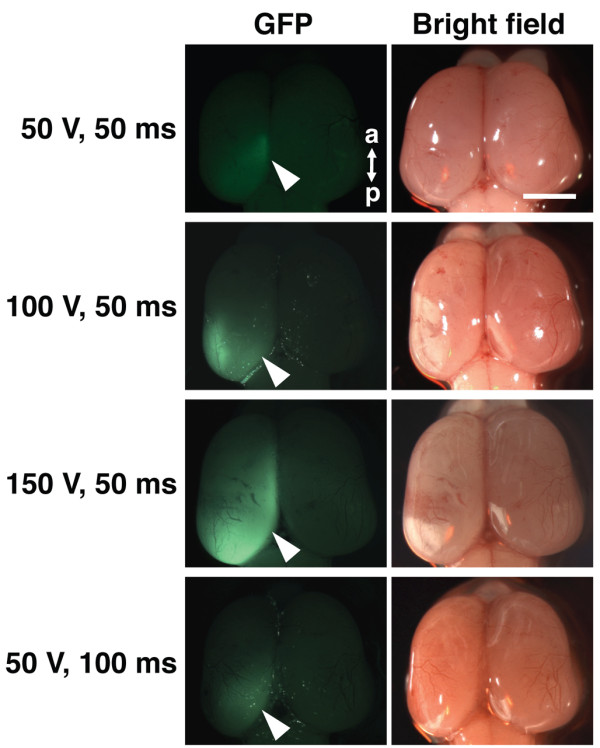
**Various parameters of* in utero *electroporation.***In utero* electroporation was performed at E36-37 to introduce pCAG-GFP under the following range of various parameters: pulse voltage 50-150 V, pulse duration 50-100 ms. Ferret embryos were dissected out 3 days later. Macroscopic dorsal views of the ferret brain (right) and GFP fluorescence (left, arrowheads) are shown. Note that higher voltage resulted in stronger GFP fluorescence, while longer pulse duration did not. a, anterior; p, posterior. Scale bar, 2 mm.

To increase the transfection efficiency, we increased either the voltage or the duration of pulses. Interestingly, when we increased the voltage of pulses up to 150 V, we found GFP-positive area and GFP-fluorescence intensity dramatically increased (Figure 3). The size of the cerebral cortex on the electroporated side was indistinguishable from that on the other side, suggesting that expressing GFP does not have any apparent adverse effects. In contrast, when we increased the duration of pulses up to 100 ms, the GFP-positive area and GFP-fluorescence intensity did not seem to be improved. These results indicate that higher voltage is needed for *in utero* electroporation for ferrets compared with that for mice. This is presumably because the sizes of the uterus and the embryo in ferrets are larger than those in mice.

So far, we had examined GFP fluorescence using embryos before birth. Because our surgical procedures and electric pulses could cause damages to embryos, we next examined whether electroporated ferret babies can be born. We performed *in utero* electroporation, and let ferret mothers deliver and raise the transfected babies. We found that ferret babies were born at the due date. We also calculated the survival rates of electroporated embryos. We performed *in utero* electroporation between E35-E38 and examined how many transfected babies were born alive. Our examination showed that higher voltage resulted in lower survival rates (50 V, 100%, n = 9; 100 V, 85%, n = 48; 150 V, 65%, n = 23).

**Figure 4:**
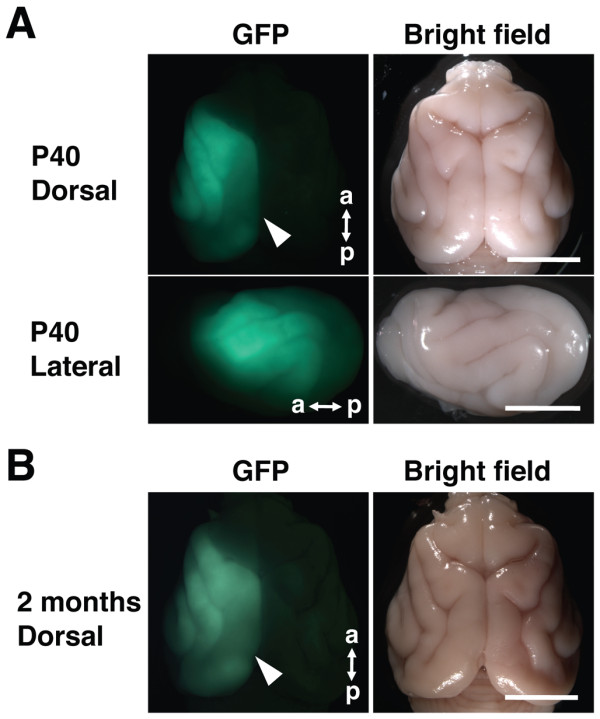
**GFP fluorescence in the postnatal ferret brain.***In utero* electroporation was performed at E35 to introduce pCAG-GFP in the cerebral cortex, and ferret mothers were allowed to raise their electroporated babies. 40 days (**A**) or 2 months (**B**) after birth, the brain was dissected out, and GFP fluorescence and bright field images were taken. Note that GFP signals were distributed over a wide range of the cortex (arrowheads), and that GFP signals were clearly visible even 2 months after birth. Dorsal, dorsal view; Lateral, lateral view; a, anterior; p, posterior. Scale bars, 1 cm.

Remarkably, intense GFP fluorescence was clearly observed at least 2 months after birth (Figure 4). These results indicate that our *in utero* electroporation procedure is applicable for investigating developmental events after birth, such as the formation of ODCs. Expressing shRNA constructs, optogenetic molecules (e.g. channelrhodopsin and halorhodopsin), transsynaptic tracers (e.g. WGA and WGA-Cre), neuronal activity reporters (e.g. GFP-based Ca^2+^ sensors) and activity-modifying channels (e.g. Kir2.1 and NaChBac) by using our procedure should be extremely useful for investigating the mechanisms underlying the function and development of the brain in higher mammals.

**Figure 5:**
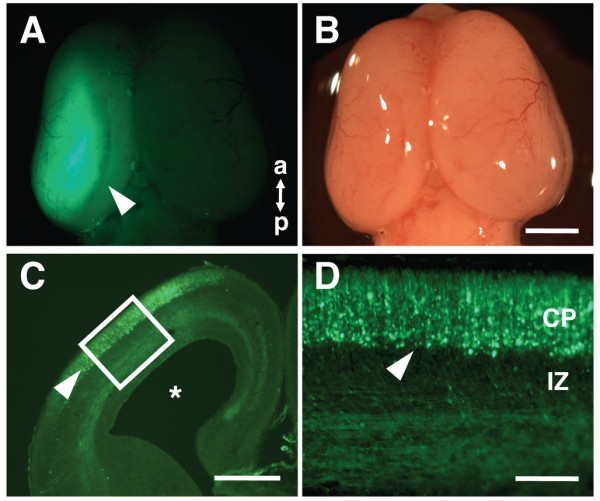
***In utero*electroporation performed at an earlier time point during development.***In utero* electroporation was performed at E31 to express GFP in the ferret cerebral cortex, and the brain was dissected at P0. Macroscopic dorsal views of the ferret brain (**B**) and its GFP fluorescence (**A**, arrowhead) are shown. a, anterior; p, posterior. Low (**C**) and high (**D**) magnification images of GFP fluorescence in the cerebral cortex in the coronal section. The area within the white box is magnified and shown in (**D**). Note that numerous GFP-positive neurons are visible in the cortical plate even without GFP staining (arrowheads). Asterisk, the lateral ventricle. CP, cortical plate; IZ, intermediate zone. Scale bars, 2 mm (**B**), 1 mm (**C**) and 200 μm (**D**).

In the case of mice, it is well-known that when *in utero* electroporation is performed earlier in development, deeper neurons are transfected. For example, *in utero* electroporation performed at E15.5 leads to layer 2/3 pyramidal neurons being transfected, while that at E12.5 results in deep cortical neurons being transfected. Therefore, we tested whether *in utero* electroporation is applicable to different time points during development using ferrets. Because a previous report using ^3^H-thymidine showed that layer 6 neurons are born around E30 in ferrets [13], we next performed *in utero* electroporation at E31 to transfect deep cortical neurons. We successfully observed intense GFP fluorescence on the brain surface (Figure 5, A and B). Consistently, in the coronal sections, numerous deep cortical neurons were GFP-positive (Figure 5, C and D). These results suggest that *in utero* electroporation is applicable to younger embryos for transfecting deep cortical neurons.

**Figure 6:**
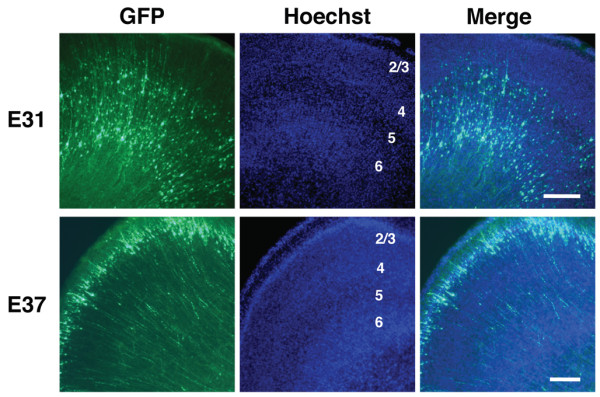
**Distribution of GFP-positive neurons in the ferret cerebral cortex.***In utero* electroporation was performed at either E31 or E37 to express GFP in the cerebral cortex, and the ferret brain was dissected out at P10-15. Coronal sections were stained with Hoechst 33342 to reveal cytoarchitectonic structures of the cortex. Cortical layers are indicated with numbers in Hoechst images. When *in utero* electroporation was carried out at E31, GFP-positive neurons were preferentially found in layers 5 and 6 (upper panels). In contrast, *in utero* electroporation at E37 resulted in layer 2/3 being transfected (lower panels). Scale bars, 200 μm.

**Figure 7:**
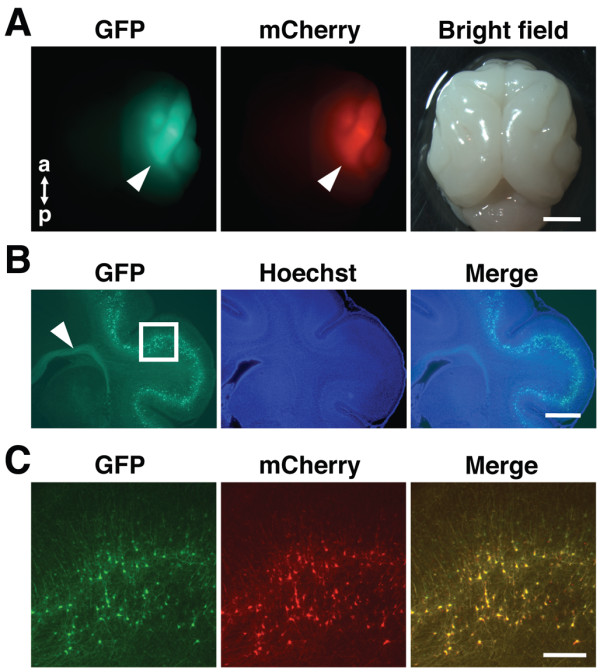
**Double labeling using*in utero*electroporation.** Cortical neurons were transfected with pCAG-GFP and pCAG mCherry using *in utero* electroporation at E33. The brain was dissected out at P20, and coronal sections were made. (**A**) GFP signals and mCherry signals were clearly observed on the brain. (**B**) Numerous GFP-positive neurons were found in deep cortical layers. GFP-positive axons were also clearly visible even without GFP immunostaining (arrowhead). The area within the white box is magnified and shown in (**C**). Note that most GFP-positive neurons were also positive for mCherry **(C)**. The morphology of GFP-positive neurons was clearly visible even without GFP immunostaining. Scale bars, 5 mm (**A**), 1 mm (**B**) and 200 μm .(**C**).

Next we examined which layers of the cerebral cortex were labeled with GFP in more detail. We performed *in utero* electroporation at E31, and the cortical sections were prepared at P15. We found that GFP-positive neurons were preferentially found in layers 5 and 6 (Figure 6). In contrast, when *in utero* electroporation was carried out at E37, most GFP-positive neurons were located in superficial layer 2/3 (Figure 6). These results are consistent with a previous report showing that deep and superficial cortical neurons are generated around E30 and birth, respectively, in ferrets [13]. Thus, our results indicate that *in utero* electroporation can be used to target most of the cortical excitatory neurons. It should be noted that the morphologies and axonal trajectories of transfected neurons were clearly visible even without using GFP immunostaining (Figures 6, 7B and 7C), suggesting that the morphological changes of axons and dendrites can be examined in living ferrets.

In addition to labeling with GFP, it would be extremely useful if multiple genes could be co-transfected using *in utero* electroporation. Therefore, we examined co-transfection efficiency by using pCAG-GFP and pCAG-mCherry. We co-transfected GFP and mCherry and dissected the brain at P20. We found bright fluorescence of both GFP and mCherry on the brain surface (Figure 7A). In coronal sections, numerous GFP-positive neurons were distributed in deep cortical layers (Figure 7B). GFP-positive axons were also clearly visible (Figure 7B, arrowhead). Then, we examined co-localization of GFP and mCherry at the cellular level using high magnification images and found that most of GFP-positive neurons were also positive for mCherry (92.2% ± 4.4, 6 sections from 3 animals) (Figure 7C). These results suggest that the co-transfection efficiency of *in utero* electroporation is reasonably high, and therefore *in utero* electroporation is a powerful means to express GFP plus either shRNA, optogenetic molecules, transsynaptic tracers, neuronal activity reporters or activity-modifying channels in higher mammals.

## Discussion

Previous pioneering works demonstrated that postnatal electroporation was feasible for expressing transgenes into neonatal ferret babies [14,15]. Gene expression resulting from postnatal electroporation, however, was restricted to superficial layer 2/3 in the ferret cerebral cortex, presumably because most cortical neurons had already been born and moved into the cortical plate from the ventricular zone when electroporation was performed postnatally. In contrast, our *in utero* electroporation procedure described here can be used to express genes of interests into a variety of cortical layers. Taken together with a previous study reporting neonatal electroporation, our study indicates that most of the cortical layers of higher mammals can be now targeted using electroporation. According to the results obtained using rodents [16-21], theoretically it should be plausible to express genes not only in excitatory neurons in the cerebral cortex but also in various brain regions such as the hippocampus, the thalamus, the retina and the amygdala. Indeed, a previous pioneering study established a procedure of postnatal electroporation for expressing multiple transgenes in the ferret retina [15].

More than 6 ferret babies are usually born from one pregnant ferret mother. This large number of ferret babies per pregnant mother relative to other higher mammals is an important advantage of ferrets. This enables us to examine various experimental conditions and to obtain a sufficient number of experimental samples. On the other hand, presumably because the ferret uterus and embryos are bigger than those of mice, transmitted light should be applied to visualize the location and shape of ferret embryos. Further, higher voltage should be used to introduce plasmids efficiently in ferrets. In addition, because the placenta is also bigger in ferrets than in mice, it seems reasonable to be careful to place glass micropipettes and electrodes far from the placenta to avoid unnecessary damage of the placenta.

Higher mammals have highly developed unique brain structures, such as ODCs, the layered dorsal lateral geniculate nucleus (dLGN), and the gyrus and OSVZ of the cerebral cortex. Although these structures have been believed to be important for the function and development of the brain, the molecular mechanisms underlying the formation of these structures and their functional significance are still elusive. This is mainly because rapid and efficient genetic methods for expressing genes into the brains of higher mammals are still poorly available. Therefore, our *in utero* electroporation method for ferrets should open the door to the next generation of neuroscientific experiments using higher mammals. Gain-of-function and loss-of-function studies using higher mammals are now ready to be launched.

It seems possible to make transgenic ferrets using virus vectors because the successful application of virus vectors to make transgenic monkeys and marmosets was reported [22-24]. Compared with virus vectors, *in utero* electroporation has several advantageous features. First, it does not take a long time to obtain transfected animals. Transfected ferrets should be available within a few weeks. Second, multiple genes can easily be introduced simultaneously as shown in this study. Third, transgenes can be selectively expressed in appropriate brain regions, even without using specific promoters, by modifying the direction of electrodes and the age when electroporation is performed. Finally, if necessary, cell type-specific promoters can be utilized because a previous report showed that larger DNA fragments such as BAC could be introduced using electroporation [25]. Importantly, our results showed that GFP expression levels were high enough to examine the dendritic morphology and axonal trajectories of transfected neurons without using GFP immunostaining, suggesting that morphological changes of neurons can be examined in living ferrets. Our *in utero* electroporation procedure provides a rapid and efficient means to express genes of interest into the brains of higher mammals. Because our results indicate that *in utero* electroporation can be used not only in rodents but also in ferrets, it seems reasonable to speculate that *in utero* electroporation is applicable to other higher mammals such as primates. It would be intriguing to establish *in utero* electroporation protocols for primates.

We recently reported that newly generated Thy1S promoter is useful for labeling cortical neurons sparsely in mice [8]. It would be intriguing to combine this Thy1S promoter and our *in utero* electroporation procedure described here in ferrets. Theoretically, using our *in utero* electroporation procedure, it seems possible to express other genes in ferrets including shRNA constructs, optogenetic molecules (e.g. channelrhodopsin and halorhodopsin), transsynaptic tracers (e.g. WGA and WGA-Cre), neuronal activity reporters (e.g. GFP-based Ca^2+^ sensors) and activity-modifying channels (e.g. Kir2.1 and NaChBac) [26-30]. Recently, local microcircuits within the cerebral cortex have been extensively investigated using new physiological techniques such as multiple simultaneous patch recordings, laser scanning photostimulation (LSPS) and channelrhodopsin-2 assisted circuit mapping (CRACM) [31-36]. Combining these techniques with *in utero* electroporation in ferrets would contribute toward an understanding of the function and structure of the brains of higher mammals.

## Methods

### Animals

Normally pigmented, sable ferrets (*Mustela putorius furo*) were purchased from Marshall Farms (North Rose, NY). Ferrets were maintained as described previously [1,2]. The day of birth was counted as postnatal day 0 (P0). All procedures were performed in accordance with a protocol approved by the University of Tokyo Animal Care Committee.

### *In utero* electroporation procedure for ferrets

We established our procedure of *in utero* electroporation for ferrets by modifying that for rodents [7,8]. Pregnant ferrets were anesthetized with sodium pentobarbital, and their body temperature was monitored and maintained using a heating pad. The uterine horns were exposed and kept wet by adding drops of PBS intermittently (Figure 1B). The location of embryos was visualized with transmitted light delivered through an optical fiber cable (Figure 1C). It is important to use transmitted light for visualizing embryos in the uterus (Figure 1D). The pigmented iris was visible, and this enabled us to assume the location of the lateral ventricle. Approximately 2-5 μl of DNA solution (0.5-1 mg/ml) was injected into the lateral ventricle at the indicated ages using a pulled glass micropipette. Because the position and shape of the placenta in ferrets are more obscure compared with those in mice, care should be taken not to damage the placenta with glass micropipettes. Each embryo within the uterus was placed between tweezer-type electrodes with a diameter of 5 mm (CUY650-P5; NEPA Gene, Japan). Square electric pulses (50-150 V, 50 ms) were passed 5 times at 1 s intervals using an electroporator (ECM830, BTX). Higher voltages resulted in higher transfection efficiency. Care was taken to quickly place embryos back into the abdominal cavity to avoid excessive temperature loss. The wall and skin of the abdominal cavity were sutured, and the embryos were allowed to develop normally. Experiments were repeated at least three times and gave consistent results.

### Plasmids

pCAG-GFP and pCAG-mCherry were described previously [7]. Plasmids were purified using the Endofree plasmid Maxi kit (Qiagen, Valencia, CA). Prior to *in utero* electroporation procedures, plasmid DNA was diluted to 0.5-1.0 mg/ml in 1xPBS, and Fast Green solution was added to a final concentration of 0.5% to monitor the injection. For co-transfection, the mixture of pCAG-GFP and pCAG-mCherry was used. Higher concentrations of plasmids would be useful, especially when the mixture of several plasmids is transfected.

### Preparation of sections

Preparation of sections was performed as described previously with modifications [37,38]. Briefly, ferrets were deeply anesthetized with pentobarbital and transcardially perfused with 4% paraformaldehyde (PFA), and then the brain was dissected. Alternatively, brains were taken from deeply anesthetized ferrets, and immersion-fixation was performed using 4% PFA. Then the brains were cryoprotected by overnight immersion in 30% sucrose and embedded in OCT compound. Sections of 50 μm thickness were incubated with 1 μg/ml Hoechst 33342, washed and mounted. Experiments were repeated at least three times and gave consistent results.

## Abbreviations

ODC, Ocular dominance column; OSVZ, Outer subventricular zone; dLGN, Dorsal lateral geniculate nucleus.

## Competing interests

The authors declare no conflict of interest.

## Authors’ contributions

HK designed research; HK, LI and KT performed research; and HK wrote the paper. All authors read and approved the final manuscript.
